# Multifunctional PLGA nanosystems: enabling integrated diagnostic and therapeutic strategies

**DOI:** 10.3389/fphar.2025.1670397

**Published:** 2025-09-18

**Authors:** Yue Li, Tao Tao, Yao Xiong, Weiyu Guo, Yangbiao Liang

**Affiliations:** Ultrasound Medical Center, Zhujiang Hospital of Southern Medical University, Guangzhou, China

**Keywords:** PLGA, theranostics, nanosystems, application, biocompatibility

## Abstract

In the past decades, biodegradable polymers have been widely used in pharmaceutical and medical engineering materials. Poly (lactic-co-glycolic acid) (PLGA) copolymer, renowned for its exceptional biocompatibility, inherent non-toxicity, and superior encapsulation and film-forming capabilities, has been widely acknowledged as one of the foremost candidate materials among next-generation biodegradable polymers with remarkable application potential. PLGA nanoparticles exhibit demonstrated versatility in accommodating hydrophobic or hydrophilic substances, which can be either encapsulated within their core matrix or adsorbed onto the surface. This includes chemical drugs, nucleic acids, peptides, and proteins. Upon entering the systemic circulation, the size-tunable characteristics of PLGA nanoparticles synergize with surface ligand-receptor interactions to confer dual-targeting capabilities: passive targeting through enhanced permeability and retention (EPR) effects, and active targeting *via* specific molecular recognition at pathological sites. Moreover, the integration of multimodal imaging capabilities into PLGA-based nanoparticles enables *in vivo* imaging-guided drug delivery, which paves the way for more precise and enhanced approaches to disease diagnosis and therapeutic intervention. This review systematically examines the fabrication strategies, structural variants of PLGA-based nanostructures, and their applications in both diagnostic and therapeutic domains of biomedicine.

## 1 Introduction

The efficacy of a drug is not only related to pharmacokinetics, but more importantly depends on its bioavailability at the intended site of action, which in turn is determined by the drug delivery method (Agnihotri et al., 2004; Allen and Cullis, 2004; Alavi et al., 2024). Medical nanotechnology is an emerging multifunctional platform, which may greatly affect the delivery of multiple therapeutic drugs, including small molecule chemicals, peptides, genes and diagnostic imaging agents (Farokhzad and Langer, 2006; Shi et al., 2016; Li et al., 2021). Nanostructure based carriers can change the treatment mode of diseases through drug delivery controlled by time and space. These therapeutic agents are encapsulated in the nanocarrier or covalently grafted on the surface of the nanocarrier. After intravenous injection, Their release is affected by carrier composition, pH of microenvironment, temperature, external regulation and other factors (Kamaly et al., 2012; Kamaly et al., 2016; Zheng et al., 2017; Yang et al., 2024a). A diverse array of materials encompassing synthetic polymers (e.g., PLGA and polyamides), natural polymers (including hyaluronic acid, chitosan, and albumin), phospholipids, along with inorganic substrates (gold, copper, silver, and zinc) have been extensively employed in the fabrication of nanocarriers (Dykman and Khlebtsov, 2016; Lee et al., 2016; Hong and Choi, 2017; Lee et al., 2017; Oberli et al., 2017; De Matteis et al., 2018; Lee and Cho, 2018; Koo et al., 2019; Beach et al., 2024). Among these different materials, PLGA is the most promising material and the preferred material for preparing drug delivery nanoparticles (Danhier et al., 2012; Martins et al., 2017; Swider et al., 2018). Table 1 presents a comparative analysis of PLGA and other nanocarriers for drug delivery. PLGA has a long history in medicine as a biodegradable suture material. PLGA can be degraded into lactic acid (LA) and Glycolic acid (GA), thus entering the metabolic pathway, and finally into water and carbon dioxide. The U.S. Food and Drug Administration (FDA) has granted regulatory approval for PLGA as a matrix material in parenteral dosage forms, solidifying its status as a pharmaceutically validated excipient for injectable formulations (Mir et al., 2017). Surface chemical modification of PLGA nanoparticles can confer biological targeting functionality and prolong their systemic circulation time. Pharmaceuticals, peptides, and nucleic acids with distinct physicochemical properties can be either encapsulated within the PLGA nanoparticle core or adsorbed onto their outer surface. Furthermore, imaging agents may be incorporated into PLGA nanoparticles to endow them with diagnostic imaging capabilities for precise localization of pathological lesions (Danhier et al., 2010; Ratzinger et al., 2010; Acharya and Sahoo, 2011; Maeda et al., 2013; Sah et al., 2013; Nottelet et al., 2015; Peres et al., 2017; Hamdallah et al., 2024). The historical progression of PLGA-based nanosystems represents a remarkable evolution in drug delivery technology. Research on biodegradable polymers for drug delivery has been ongoing since they were first used as bioresorbable surgical devices in the 1980s. The progression from conventional drug delivery to multifunctional diagnostic-therapeutic applications represents a significant advancement in PLGA nanotechnology. Recent developments have demonstrated innovative approaches such as the anti-VEGFR2 antibody-coated PLGA nanoparticles for ocular delivery combine photothermal and chemotherapy to treat corneal neovascularization, and PLGA-PEG nanoparticles encapsulating DOX, SPIONs exhibit superior cytotoxicity, reduced cardiotoxicity, dual-mode MRI-fluorescence imaging (Salmasi et al., 2024; Ye et al., 2024). These advancements highlight the evolution of PLGA nanosystems from simple drug carriers to sophisticated theranostic platforms that combine targeted therapy with diagnostic capabilities. The simplicity and reliability of the PLGA nanoparticles fabrication techniques, coupled with their excellent biocompatibility, have significantly enhanced the feasibility of PLGA-based nanoparticles in clinical applications. This paper elucidates the fundamental principles of employing PLGA as a drug delivery vehicle, outlines fabrication methodologies for PLGA-based nanostructures, and examines the versatile applications of these nanopolymeric systems across diverse pathologies including cardiovascular disorders, neurodegenerative diseases, infectious conditions, inflammatory ailments, with particular emphasis on their implementation in the diagnosis and treatment of malignancies.

**TABLE 1:** Comparative analysis of PLGA vs other nanocarriers in drug delivery.

Feature	PLGA	Liposomes	Dendrimers	Inorganic nanoparticles
Material Composition	Synthetic biodegradable polymer (lactic/glycolic acid)	Phospholipid bilayers (often with cholesterol/PEG lipids)	Highly branched synthetic macromolecules (e.g., PAMAM)	Gold, silver, mesoporous silica*etc.*
Size Range	Nano- to micro-scale (often 100–300 nm)	Typically 50–200 nm	Molecularly precise (1–10 nm)	Variable (e.g., gold: 10–100 nm; silica: up to hundreds of nm)
Drug Loading	High for hydrophobic/hydrophilic drugs (Yang et al., 2024b)	High for hydrophilic (core) and hydrophobic (lipid bilayer) drugs	Moderate, limited by generation and internal cavities	Variable (mesoporous silica: high; gold: low, often surface-conjugated)
Release Profile	Sustained release (days to weeks) *via* diffusion/degradation	Biphasic (initial burst + sustained release)	Rapid release, modifiable *via* surface functionalization	Stimuli-responsive (e.g., light, heat, magnetic)
Targeting Ability	Requires surface functionalization for active targeting	Easily functionalized for active targeting	Excellent for active targeting due to multifunctional surface groups	Easily functionalized; strong active targeting potential
Biocompatibility	Excellent; biodegradable (FDA-approved); metabolites are safe (Kakavandi et al., 2025)	High (membrane-like); but some cationic lipids may show toxicity	Generation-dependent; high generations may cause membrane toxicity	Poor biodegradation; potential long-term toxicity (e.g., metal accumulation)
Clinical Progress	Widely used; multiple FDA-approved products (e.g., implants, microspheres)	Several clinical products (e.g., Doxil^®^ for chemotherapy) (Gabizon et al., 2025)	Mostly preclinical; some early-phase clinical trials	Few clinical applications (e.g., AuroShell^®^ for photothermal therapy) (Chen et al., 2023)
Key Advantages	Proven safety, tunable degradation, sustained release, versatile delivery routes	High biocompatibility, dual drug loading, mature technology	Precise structure, monodispersity, high surface functionality	Unique properties (optical, magnetic, electronic) for theranostics
Major Limitations	Burst release, organic solvent use in preparation, batch-to-batch variability	Stability issues (leakage), complement activation (CARPA)	Complex synthesis, high cost, scalability challenges	Poor biodegradability, potential long-term toxicity

## 2 The principle and preparation method of PLGA as a drug nanocarrier

Modern medicine asserts that pathological conditions originate from aberrant cells within bodily organs, and effective therapeutic intervention fundamentally relies on precise delivery of pharmacological agents. To achieve successful localization at diseased cells, these therapeutic compounds must overcome multiple physiological barriers including inadequate solubility, suboptimal membrane permeability, and rapid metabolic degradation. Implementation of advanced drug delivery systems designed for targeted transport to pathological cellular sites can simultaneously enhance therapeutic safety profiles, improve clinical efficacy, and mitigate intrinsic pharmaceutical limitations such as systemic toxicity, compromised hydrophilicity, molecular instability, and insufficient tissue specificity. Medical nanotechnology innovations have significantly advanced the engineering of nanoscale therapeutic delivery platforms. The polymer PLGA, certified by the FDA for its biodegradability, has become a cornerstone material in fabricating these precision-targeted nanocarriers, enabling enhanced pharmacological agent localization and temporally regulated payload release. At present, PLGA nanoparticles carrying various hydrophilic and hydrophobic drugs are being studied to solve various thorny diseases. In addition, many studies are exploring various synthesis techniques to improve the targeting ability of PLGA nanoparticles and enable them to be highly specific in transporting to target organs (Matoba and Egashira, 2014). The preparation method of PLGA nanoparticles may affect their physical and chemical properties, such as particle size, particle shape, particle size distribution, suspension stability, and drug encapsulation efficiency (Allahyari and Mohit, 2015). The methods for preparing PLGA nanoparticles include the following: (1) Emulsion evaporation method, which is the most commonly used method, divided into single emulsion method and double/multiple emulsion method. The single emulsion method begins by solubilizing PLGA and hydrophobic drugs in organic media (e.g., chloroform). This organic phase is subsequently combined with a surfactant-stabilized aqueous solution (typically containing polyvinyl alcohol) to generate an oil-in-water dispersion. Mechanical homogenization or ultrasonic energy is then applied to achieve nanoemulsion formation. PLGA nanoparticles can be obtained through centrifugation, washing, and freeze-drying (Mir et al., 2017; Swider et al., 2018). The double/multiple emulsion approach initiates by incorporating an aqueous suspension of hydrophilic therapeutic agents into a PLGA-containing matrix, creating a primary water-in-oil dispersion. This intermediate emulsion is subsequently dispersed into a secondary surfactant-rich aqueous medium, establishing a stabilized multi-compartmental emulsion system. Through precisely controlled solvent elimination, structurally consolidated nanoparticles are obtained. This versatile methodology enables simultaneous encapsulation of both lipophilic and hydrophilic pharmaceutical compounds. Furthermore, colloidal stabilization and dimensional characteristics can be modulated by optimizing mechanical agitation parameters such as rotational velocity and energy input during the emulsification process (Rezvantalab et al., 2018). (2) Microfluid method, microfluidic systems represent an emerging approach for precise control of minute fluid quantities. This methodology operates on the fundamental concept where two immiscible fluids converge within micron-sized channels. Through the application of external forces or hydrodynamic shear stress, these fluids fragment into nanoscale emulsion droplets. Subsequent solvent evaporation induces particle solidification, ultimately yielding uniform nanoparticles. The advantages of microfluidic methods are uniform particle size distribution, high repeatability, and easy surface modification. Its limitation is that the production scale is small and microchannels are prone to blockage (Sun et al., 2014; Feng et al., 2015). (3) Nanoprecipitation method, PLGA polymers and hydrophobic therapeutic agents demonstrate mutual solubility in water-miscible organic solvents. The resultant homogeneous organic phase undergoes controlled-rate infusion into a surfactant-stabilized aqueous phase *via* precision syringe pumping. Subsequent rapid solvent diffusion initiates interfacial phase separation, driving the spontaneous self-assembly of PLGA-drug complexes into monodisperse nanoparticles. This nanoprecipitation methodology exhibits exceptional reproducibility and operational simplicity, while its inherent hydrophobicity-driven assembly mechanism inherently limits encapsulation efficacy for hydrophilic pharmaceutical compounds (Ghaly et al., 2025; Long et al., 2025). (4) Spray-drying method, hydrophilic drug-loaded aqueous phases can be homogenized with PLGA-containing organic phases under high-shear conditions to generate stable water-in-oil emulsions, which may subsequently be engineered into complex multiple emulsion architectures. Particulate matrices are obtained through subsequent implementation of thermally assisted aerodynamic atomization, where controlled pneumatic dispersion of the emulsion systems is coupled with rapid solvent evaporation under optimized thermal conditions (Booysen et al., 2013). Despite its demonstrated versatility in hydrophobic drug encapsulation, spray-drying techniques exhibit critical operational constraints primarily stemming from wall deposition phenomena. Particulate adhesion to internal reactor surfaces during the aerodynamic atomization process substantially compromises production yields, necessitating systematic process parameter optimization to mitigate interfacial interactions between nascent nanoparticles and containment substrates (Ding and Zhu, 2018). A critical comparison of PLGA nanoparticle fabrication techniques, focusing on scalability, reproducibility, cost, and clinical suitability, is summarized in Table 2.

**TABLE 2:** Critical comparison of PLGA nanoparticle fabrication techniques.

Fabrication method	Scalabi-lity	Reprodu-cibility	Cost-effective-ness	Suitability for clinical translation	Advantages	Limitations
Emulsion Evaporation (Swider et al., 2018)	High	Moderate	Moderate (solvent removal adds cost)	High (well-established, scalable)	Versatility for hydrophobic/hydrophilic drugs	Broad size distribution; use of hazardous solvents
Microfluidics (Feng et al., 2015)	Low	High	Low (low throughput, high equipment cost)	Low (currently limited to pre-clinical scale)	Excellent size control, high monodispersity	Low production scale; channel clogging
Nanoprecipitation (Ghaly et al., 2025)	Moderate	High	High (simple, low energy input)	Moderate to High (simple process, good for lipophilic drugs)	Operational simplicity, high reproducibility	Unsuitable for hydrophilic drugs
Spray-Drying (Booysen et al., 2013)	High	Moderate	Moderate (yield loss from wall deposition increases cost)	High (continuous production potential)	Rapid, single-step, continuous process	Particle adhesion to reactor walls, reducing yield

## 3 The application of PLGA in cardiovascular diseases

**FIGURE 1:**
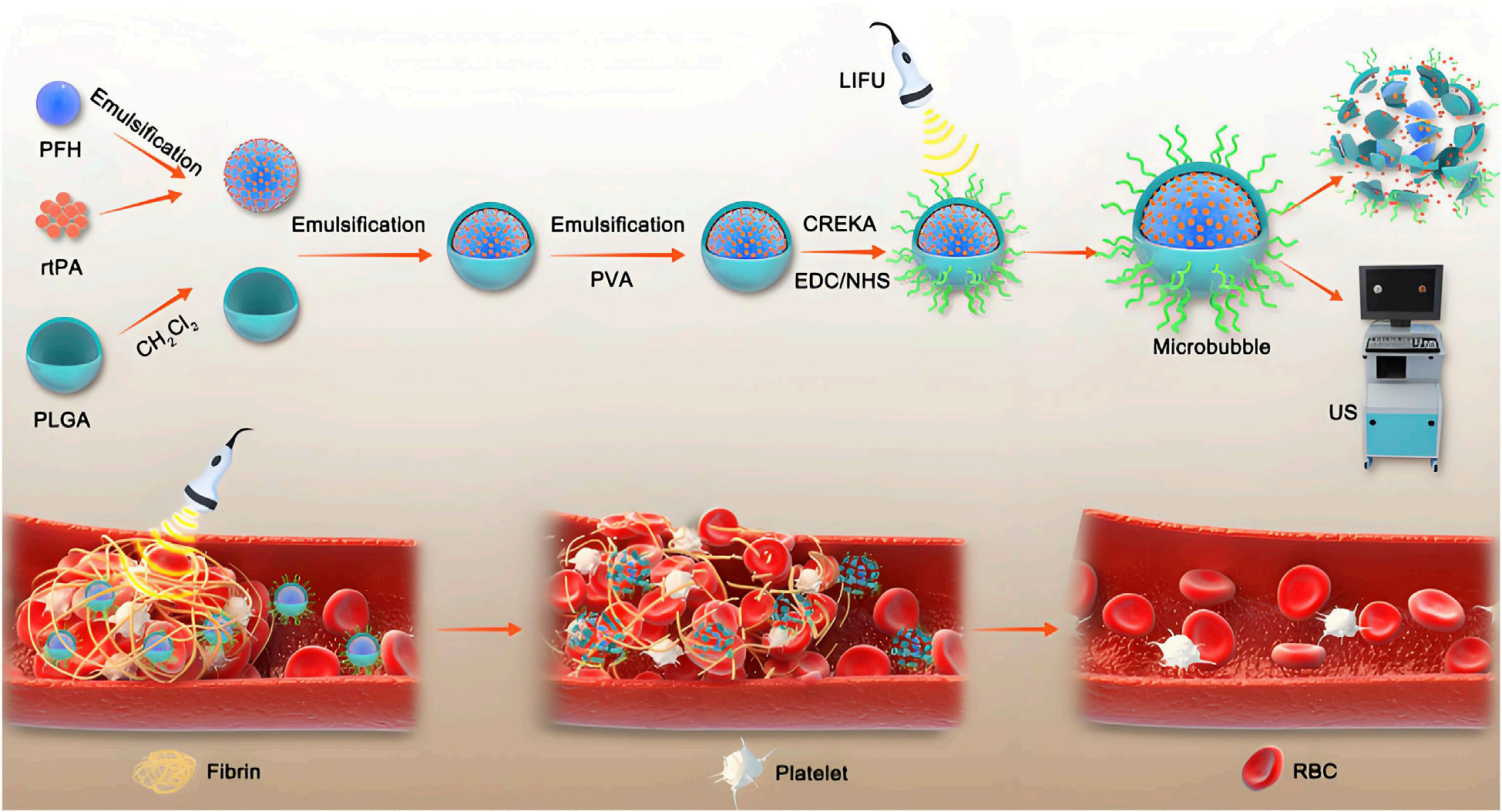
This study developed a thrombus-fibrin-targeting phase-change nanoparticle (NP) with a core composed of perfluorohexane (PFH) and the thrombolytic drug rtPA, surface-modified with CREKA peptide. The results demonstrated that under low-intensity focused ultrasound (LIFU) triggering, the NP enabled precise release of rtPA and synergized with the mechanical disruption of thrombus structures through PFH’s “liquid-gas” phase transition effect (Hu L. et al., 2022).

Cardiovascular disease is the main cause of morbidity and mortality in developed and developing countries. It is estimated that 18 million people die each year, with cardiovascular diseases accounting for 32% of global deaths (Lloyd-Jones et al., 2010; Tucka et al., 2011; Barkas et al., 2024). With further understanding of the pathological mechanisms and causes of cardiovascular diseases, coupled with the rapid development of material engineering and biotechnology, drug delivery systems based on PLGA nanoplatforms are being applied to the treatment of cardiovascular diseases. The main focus of nanotechnology application in cardiovascular research is atherosclerosis, myocardial infarction and thromboembolism (Hu Q. et al., 2022; Katsuki et al., 2022; Dash et al., 2023; Wu et al., 2024). Atherosclerosis is a chronic inflammatory vascular disease characterized by gradual thickening of the arterial wall (Libby et al., 2002; Bäck et al., 2019; Soehnlein and Libby, 2021). Small molecule drugs, such as statins, are commonly used to reduce atherosclerosis (Adhyaru and Jacobson, 2018). However, there are many issues with the systemic administration of these chemicals, including poor bioavailability, slow therapeutic effects, and serious side effects. In order to overcome these problems, PLGA was used to synthesize various nanocarriers to deliver small molecule drugs targeting atherosclerotic plaque. The specific release of such drugs at the lesion can improve their bioavailability and therapeutic efficacy (Chan et al., 2017; Cheng et al., 2018; Kim et al., 2018; Zhou et al., 2018). The PLGA nanoparticles loaded with pitavastatin prepared by Katsuki et al. inhibit monocyte recruitment through low expression of chemoattractant proteins and their stimulating factors during sustained release of statins, thereby reducing plaque instability and rupture (Katsuki et al., 2014). Anning Yanget al. Developed PLGA hybrid nanocomplexes co-loaded with Pitavastatin and Resveratrol, targeting macrophage ferroptosis to reduce lipid deposition and inflammation in atherosclerosis. It demonstrated significant plaque reduction *in vivo*, offering a novel strategy for atherosclerosis treatment (Yang et al., 2025). Imatinib is a platelet derived growth factor receptor (PDGFR) inhibitor. The activity of PDGFR has a serious impact on the progress of atherosclerosis. Stabilin-2 is a glycoprotein that widely exists in atherosclerotic plaque. S2P is a peptide that selectively binds to Stabilin-2. Imatinib loaded PLGA nanoparticles modified with S2P peptide are used to deliver targeted drugs to atherosclerotic plaque (Esfandyari-Manesh et al., 2020). In addition to targeted and specific release, another major obstacle to the application of nanomedicines is that most nanoparticles are cleared by the immune system before reaching the target (Blanco et al., 2015; Liu et al., 2017). To better evade immune system clearance and release drugs at plaque sites, different biomimetic designs have emerged (Wei et al., 2017; Martinez et al., 2018; Boada et al., 2020; Park et al., 2020). In the early stage of atherosclerosis, activated endothelial cells induce macrophage recruitment by secreting adhesion molecules and chemokines. Therefore, macrophage based biomimetic nanoparticles can precisely target the inflammatory region in atherosclerotic lesions. Wang et al. covered the surface of rapamycin loaded PLGA nanoparticles with a macrophage membrane coating, which can be accumulated in activated endothelial cells, It can effectively inhibit the phagocytosis of macrophages and the progression of atherosclerosis *in vivo* (Wang et al., 2021). Erythrocyte membrane has high biocompatibility, EPR and long half-life. PLGA coated with erythrocyte membrane can accurately deliver rapamycin to atherosclerotic plaque to reduce the progression of atherosclerosis (Wang Y. et al., 2019). In myocardial ischemia, myocardial cell death and fibrosis can lead to impaired myocardial function. Proangiogenic cytokines such as vascular endothelial growth factor (VEGF) and fibroblast growth factor (FGF) can stimulate neovascularization in ischemic heart tissue and improve functional impairment (Maija et al., 2014; Ghalehbandi et al., 2023; Morita and Hoshiga, 2024; Voronkov et al., 2024). PLGA nanoparticles loaded with VEGF have been shown to be a promising cytokine delivery system in rat myocardial ischemia models (Formiga et al., 2010). Yokoyama et al. demonstrated that PLGA nanoparticles loaded with simvastatin can significantly enhance cell migration and growth factor expression *in vitro*, and target mouse myocardial ischemic regions and induce endogenous heart regeneration through intravenous administration (Yokoyama et al., 2019). Coronary artery reperfusion leads to abnormal death of myocardial cells, known as myocardial ischemia-reperfusion injury. Monocytic inflammatory mediators demonstrate pivotal involvement in this pathophysiological cascade, indicating that they are potential targets for treating myocardial ischemia-reperfusion injury. PLGA nanoparticles loaded with irbesartan can enhance the protection of myocardial extracellular matrix from ischemia-reperfusion injury by inhibiting monocyte recruitment in myocardial ischemia-reperfusion injury models (Nakano et al., 2016). Thrombotic events constitute a critical pathophysiological mechanism underlying potentially fatal cardiovascular disorders, particularly acute myocardial infarction, cerebrovascular ischemia, and pulmonary thromboembolism. The current thrombolytic therapy, which involves injecting plasminogen activator (PA), is still limited by narrow treatment windows, rapid drug elimination, and the risk of bleeding (Rashedi et al., 2024). PLGA nanostructures loaded with PA can protect thrombolytic drugs from enzymatic degradation, optimize therapeutic outcomes in thrombus management through enhanced pharmacological precision while concurrently mitigating treatment-related complications. The surface PEG of PLGA nanoparticles plays an important role in biocompatibility and can improve pharmacokinetics. Colasuono et al. prepared a disk-shaped porous nanostructure using a mixture of PLGA and PEG and loaded it with rtPA. Although lacking active targeting ability, the high thrombolytic potential of these nanoparticles may be attributed to their ability to mimic the shape and deformation of red blood cells, leading to their significant accumulation at the site of thrombosis (Colasuonno et al., 2018). Stimuli-responsive PLGA-based phase-transition nanovesicles incorporating thrombolytic payloads have emerged as a novel theranostic platform. In the innovative approach developed by Liu hu et al., recombinant tissue plasminogen activator (rtPA) was encapsulated within these intelligent nanocarriers, while surface functionalization with CREKA peptide ligands conferred fibrin-specific targeting capability through selective binding to thrombus-associated fibrin networks. Upon exposure to low-intensity focused ultrasound (LIFU) irradiation, the nanovesicles undergo controlled volumetric oscillations (expansion) that induce mechanical disruption of thrombus architecture. This cavitation-mediated structural destabilization synergistically facilitates both rapid payload release and enhanced intralesional drug permeation, thereby amplifying rtPA’s fibrinolytic efficacy through dual-action mechanisms. The integration of acoustic-mechanical thrombus ablation with pharmacologically-enhanced clot dissolution establishes a synergistic therapeutic paradigm for precision thrombosis management (Figure 1) (Hu L. et al., 2022).

## 4 The application of PLGA in central nervous system drug delivery

**FIGURE 2:**
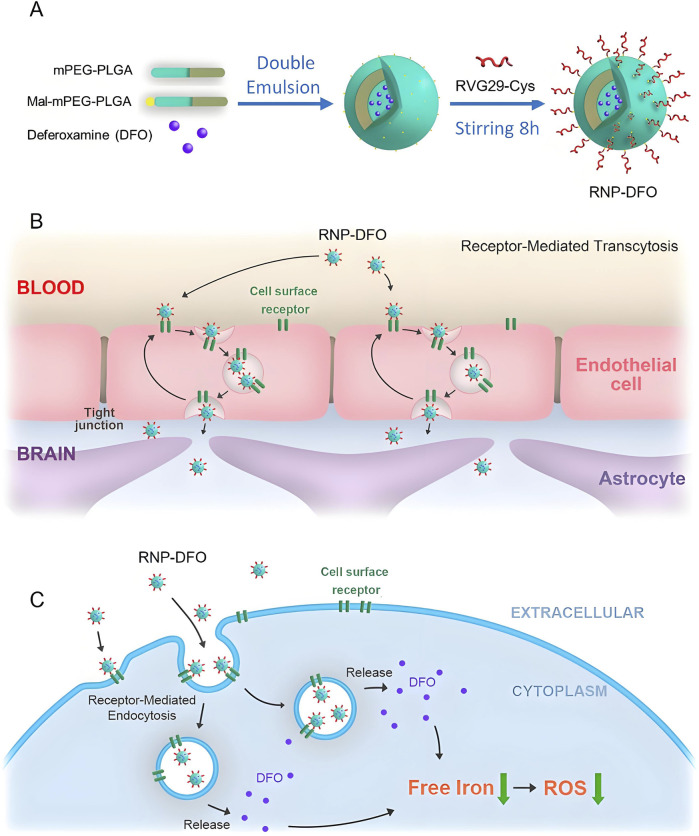
This study developed an RVG29-modified polymeric nanoparticle system for targeted delivery of deferoxamine (DFO) to treat Parkinson’s disease (PD). As shown in the panel **(A-C)**, the system penetrates the blood-brain barrier *via* RVG29-mediated receptor-mediated endocytosis, significantly reducing nigrostriatal iron deposition and oxidative stress in PD mice, alleviating dopaminergic neuron injury, and reversing neurobehavioral deficits (You et al., 2018).

The demographic aging trend worldwide has precipitated a surge in neurodegenerative disorder prevalence (Hou et al., 2019), with therapeutic intervention efficacy significantly constrained by the blood-brain barrier’s (BBB) restrictive permeability to neuropharmaceuticals. To address these pharmacological constraints and enhance therapeutic outcomes, scientific investigation increasingly focuses on PLGA-based nanoparticulate delivery vehicles. These engineered nanocarriers demonstrate promising potential for managing major neurodegenerative pathologies, including Alzheimer’s disease, Parkinson’s disease, Huntington’s chorea, amyotrophic lateral sclerosis, and multiple sclerosis, through optimized CNS-targeted drug translocation (Barbu et al., 2009; Chen and Liu, 2012; Meir et al., 2014; Saraiva et al., 2016). Alzheimer’s disease (AD) represents the predominant neurodegenerative disorder, manifesting as progressive cognitive deterioration. Its distinctive neuropathological hallmarks include extracellular aggregation of β-amyloid (Aβ) peptides and intraneuronal accumulation of hyperphosphorylated tau protein, forming neurofibrillary tangles composed of paired helical filaments (Khalid et al., 2010; Murphy et al., 2010). Curcumin exhibits anti amyloid effects by inhibiting the formation of new Aβ aggregates and clearing existing Aβ aggregates. Curcumin cannot effectively cross the BBB, resulting in lower brain intake. Therefore, PLGA nanoparticles have been developed for encapsulating curcumin to enhance its ability to cross the BBB. Fan et al. studied the encapsulation of curcumin in PLGA-PEG nanoparticles functionalized with B6 peptide and observed that compared with natural curcumin, APP/PS1 mice showed improved spatial learning and memory abilities (Fan et al., 2018). As mentioned earlier, AD is related to protein (i.e., A β) aggregation, which induces oxidative stress and leads to neuroinflammation. Thymoquinone (TQ) is a plant compound with antioxidant and anti-inflammatory properties. Yusuf et al. designed a PLGA nanoparticle surface-functionalized with polysorbate 80 loaded with thymoquinone (P-80-TQ), which acted on a mouse model of AD. The P-80-TQ nanoparticle crossed the blood-brain barrier through the endocytosis, significantly increasing superoxide dismutase (SOD) activity. After treatment with P-80-TQ nanoparticle, histopathological examination of the hippocampus tissue showed a sharp decrease in protein aggregates produced by streptozotocin induced AD in mice (Yusuf et al., 2021). Huang et al. functionalized PLGA nanoparticles with an iron-mimetic cyclic peptide (CRT) to target the transferrin/transferrin receptor protein complex, thereby enhancing BBB permeability. The spatial memory and recognition ability were significantly improved, and the levels of A β, ROS, TNF, and IL-6 in the brain of AD mice decreased (Na et al., 2017). Overall, the PLGA nanocarrier system can improve the bioavailability of the brain and ameliorate AD symptoms, reduce neuroinflammation, clear/inhibit the formation of A β aggregates, and lower the levels of several AD brain biomarkers. Parkinson’s disease (PD) manifests characteristic neuropathological hallmarks marked by progressive degeneration of dopaminergic neurons within distinct nigrostriatal pathways and the intraneuronal accumulation of Lewy bodies containing misfolded α-synuclein aggregates. The pathophysiological cascade further involves dysregulation of dopaminergic neurotransmission, mitochondrial bioenergetic deficits, redox imbalance, and chronic neuroinflammatory responses, collectively contributing to nigrostriatal system deterioration (Luk et al., 2012; Chu et al., 2023; Lababidi and Azzazy, 2025). Chen et al. prepared PLGA nanoparticles loaded with antioxidant activity puerarin for the treatment of PD, and found that they could increase the drug’s half-life and bioavailability, improve behavioral deficits in mice, and restore dopamine levels (Chen T. et al., 2019). You et al. engineered a rabies virus glycoprotein (RVG)-functionalized PLGA nanoplatform encapsulating deferoxamine mesylate, a clinically approved iron chelator, to address iron dyshomeostasis-mediated oxidative neurodegeneration in PD. This targeted delivery system demonstrated efficacy in attenuating nigrostriatal iron accumulation and redox imbalance through selective BBB penetration, thereby preserving dopaminergic neuronal integrity in substantia nigra pars compacta-striatum circuitry (Figure 2) (You et al., 2018). Bali et al. designed PLGA nanoparticles loaded with rasakin. The release of rasakin restored presynaptic dopamine consumption in the PD model. In addition, a 99 mTC loaded PLGA nanoparticle was used for gamma scintillation scanning to evaluate the permeability and targeting efficiency of the BBB, and it was observed that it can cross the BBB and target the brain (Bali and Salve, 2020). Multiple sclerosis is an autoimmune neurodegenerative disease, in which the immune system recognizes myelin sheaths as foreign antigens, leading to neuronal demyelination and gradual loss of neuronal structure and function (Trapp and Nave, 2008; Baecher-Allan et al., 2018). Leukemia inhibitory factor (LIF), a multifunctional therapeutic cytokine, exhibits dual immunoregulatory activity and myelination-enhancing potential. Rittchen et al. engineered LIF-loaded PLGA nanocarriers for targeted oligodendrocyte precursor cell (OPC) delivery, demonstrating enhanced differentiation of OPCs into mature myelinating oligodendrocytes and subsequent remyelination efficacy in demyelination models (Rittchen et al., 2015). Hunter and Gholamzad prepared PLGA nanoparticles loaded with the myelin proteolipid protein, for the treatment of multiple sclerosis mouse models. The results showed that they could reduce the neuroinflammation in the nervous system (Zoe et al., 2014; Gholamzad et al., 2020). Huntington’s disease (HD) is a hereditary disease, and its symptoms are often accompanied by progressive motor, behavioral, and mental disorders, which are related to the death of striatal neurons (Ferrante et al., 1985; David, 2002). Valenza developed cholesterol loaded PLGA nanoparticles and functionalized them with g7 glycopeptides to enhance the BBB targeting and brain accumulation of PLGA nanoparticles. This nanosystem can partially ameliorate the symptoms of the HD mice (Valenza et al., 2015). Cano used PLGA nanoparticle carrying ascorbic acid to treat HD, and the results showed reduced loss of striatal neurons (Cano et al., 2019; Cano et al., 2021). Amyotrophic lateral sclerosis (ALS), a fatal motor neuron disorder affecting spinal anterior horn cells, drives progressive neuromuscular degeneration (Pineda et al., 2024). Medina et al. engineered PEGylated PLGA nanocarriers to enhance hydrophobic drug bioavailability and pharmacokinetic profiles. This CNS-targeted formulation modulates retinoic acid receptor-mediated signaling pathways, demonstrating neuroprotective effects through improved motor function preservation and survival extension in preclinical ALS models (Medina et al., 2020). To address the side effects of high-dose riluzole in ALS treatment, Gerard Esteruelas et al. developed pVEC and PEG-functionalized PLGA nanoparticles for targeted delivery. This system enhances riluzole transport across the BBB, improves motor neuron uptake, reduces off-target accumulation, and shows promise as a targeted therapy for motor neuron diseases (Esteruelas et al., 2025).

## 5 The application of PLGA in digestive system diseases

**FIGURE 3:**
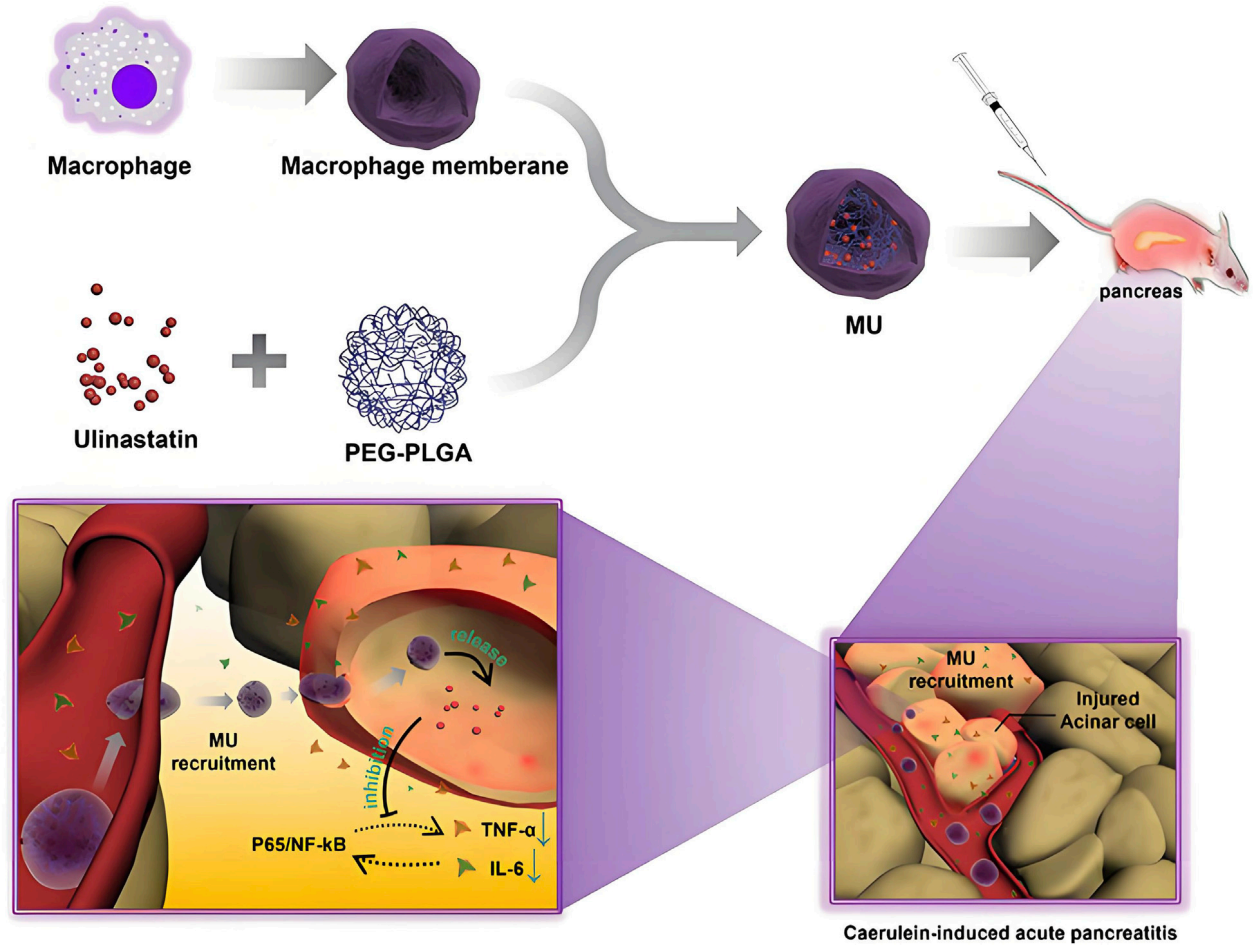
This study developed macrophage membrane biomimetic nanoparticles (MU) by encapsulating a polyethylene glycol-polylactic-co-glycolic acid-ulinastatin (PEG-PLGA-ulinastatin) composite to achieve targeted delivery. Experiments confirmed that MU exhibits excellent stability and biocompatibility, enabling precise targeting of subcutaneous and pancreatic inflammatory lesions *via* adhesion protein-mediated interactions. MU inhibits pro-inflammatory cytokine release, maintains cellular viability, and effectively alleviates AP symptoms (Yunlong et al., 2023).

The main cause of gastric ulcer is excessive secretion of gastric acid, and anticholinergic drugs, antacids, histamine H2 receptor antagonists, and proton pump inhibitors are the main treatment options. However, existing treatment modalities often face restricted implementation due to insufficient therapeutic outcomes coupled with undesirable pharmacological responses (Almadi et al., 2024; Bhatnagar et al., 2024). As a bioactive phytonutrient derived from citrus species, Diosmin demonstrates triple therapeutic potentials: modulating inflammatory pathways, neutralizing reactive oxygen species, and protecting gastrointestinal mucosa. Its pharmacological application is nevertheless challenged by inherent physicochemical limitations, particularly low hydrophilicity that leads to suboptimal drug dissolution and subsequent reduction in systemic bioavailability (Vrbata et al., 2014; Russo et al., 2018). The application of PLGA nanoparticles has shown remarkable efficacy in facilitating pharmaceutical transport across multiple physiological interfaces. These biodegradable polymeric systems exhibit unique capabilities to enhance therapeutic compound penetration through specialized biological membranes, particularly demonstrating success in traversing the neurovascular unit of the central nervous system, intestinal epithelial barriers, olfactory pathways, and corneal-endothelial interfaces (Tracy et al., 2023). Chitosan, a cationic biopolymer derived from the partial deacetylation of chitin, exhibits pH-responsive solubility and intrinsic mucoadhesive properties in acidic environments. Functionalizing PLGA nanocarriers with this amphipathic polysaccharide creates a multifunctional platform capable of stabilizing macromolecular therapeutics (e.g., proteins) through electrostatic shielding, while simultaneously conferring cationic surface characteristics *via* protonated amino groups. The reactive hydroxyl and amine moieties further enable covalent conjugation of site-specific ligands, synergistically enhancing cellular affinity and prolonging residence time at mucosal interfaces. This dual-action modification strategy optimizes drug transport kinetics across epithelial barriers, thereby achieving therapeutic efficacy at reduced dosages while mitigating systemic toxicity through localized delivery mechanisms (Chuah et al., 2011; Chronopoulou et al., 2013). A study has shown that chitosan coated PLGA nanoparticles loaded with Diosmin can improve gastric retention and cellular uptake of Diosmin in the treatment of gastric ulcers through oral administration (Abd El Hady et al., 2019). Pancreatitis is a non infectious inflammation that occurs in the pancreatic region, mainly divided into acute pancreatitis and chronic pancreatitis. Acute pancreatitis is caused by various etiologies, leading to acute damage such as pancreatic tissue edema, bleeding, and necrosis, while chronic pancreatitis represents a persistent inflammatory disorder of pancreatic parenchyma characterized by irreversible fibro-inflammatory progression, manifesting as focal or generalized glandular destruction with multifactorial etiological origins. Ulinastatin is commonly used in clinical practice to treat acute pancreatitis, but its therapeutic effect is limited by the blood pancreatic barrier and low specificity. Yunlong Chen et al. prepared macrophage biomimetic nanoparticles (MU) using macrophage membranes as the outer shell for loading ulinastatin PLGA nanoparticles. Research has found that MU exhibits good stability and biocompatibility both *in vivo* and *in vitro*. According to *in vivo* fluorescence imaging, MU demonstrates specific anti-inflammatory targeting capacity across multiple experimental paradigms, showing robust therapeutic efficacy in both hypodermal inflammatory lesions and pancreas-specific pathophysiological murine systems. Mechanistic investigations employing integrated transcriptomic-proteomic analysis reveal that MU exerts its pancreoprotective effects through coordinated modulation of inflammatory signaling cascades, particularly *via* downregulation of NF-κB-mediated cytokine storm and preservation of acinar cell mitochondrial membrane integrity (Figure 3) (Yunlong et al., 2023). The clinical application of therapeutic peptides often faces challenges in absorption, distribution, metabolism, and excretion. The use of somatostatin (SST) peptide in the treatment of chronic pancreatitis is limited by its short half-life and potential side effects. Therefore, Fang Wang et al. developed a PLGA nanocarrier system coated with macrophage membrane to improve the bioavailability of SST. Compared with macrophages, membrane polymer hybrid nanoparticles have higher cellular activity, are more easily ingested by activated endothelial cells, and exhibit better biocompatibility and targeting ability *in vitro*. Following systemic administration in chronic pancreatitis murine models, engineered biohybrid nanocarriers demonstrated multifunctional therapeutic outcomes, including elevation of somatostatin bioavailability in circulatory and pancreatic compartments, suppression of fibro-inflammatory mediators (TGF-β1, IL-6), and attenuation of histopathological damage indices. The optimized pharmacological profile of SST validates the synergistic advantages of PLGA-biomembrane conjugation in developing precision peptide delivery architectures. This modular nanosystem platform exhibits translational extensibility for diverse therapeutic peptides, enabling targeted biodistribution while addressing formulation challenges related to proteolytic stability and epithelial permeability, thereby advancing peptide-based pharmacotherapy in clinical settings (Wang et al., 2022). Inflammatory bowel disease (IBD) represents a category of immune-mediated relapsing-remitting disorders affecting the gastrointestinal tract, clinically classified into two distinct entities: ulcerative colitis (UC) characterized by continuous colonic mucosal inflammation, and Crohn’s disease (CD) marked by discontinuous segmental transmural lesions. Both subtypes exhibit cyclical disease activity patterns with alternating phases of inflammatory exacerbation and quiescence, necessitating localized therapeutic intervention through site-targeted delivery systems to achieve mucosal barrier restoration and sustained disease control (Ashton and Beattie, 2024; Triantafillidis et al., 2024). Kohei Tahara et al. developed a chitosan (CS) modified PLGA nanoparticle oral delivery system loaded with nuclear factor kappa B (NF kB) bait oligonucleotide (ODN) to evaluate its application in UC experimental models. The results showed that the system improved the stability of ODN towards DNase I and gastric juice, significantly enhancing cell uptake, effectively improving dextran sulfate induced diarrhea, reducing bloody stools, and lowering myeloperoxidase activity. By using a nano targeted drug delivery system with specific accumulation ability in the inflammatory mucosal area, pharmacotherapeutic optimization in IBD management through targeted delivery systems significantly attenuates treatment-related toxicities arising from systemic immunomodulatory regimens (Tahara et al., 2011). The aggregation and deposition of different functionalized PLGA nanoparticles on the surface of polyethylene glycol in inflamed mucosa increase, which is considered an innovative strategy for treating IBD (Hua et al., 2015). Cecília Cristelo et al. developed ROS-responsive cleavable PEG-functionalized PLGA nanoparticles for targeted oral delivery of budesonide to inflamed intestines. These nanoparticles enhance drug retention, reduce cytokine release, and significantly alleviate inflammation in colitis mice, demonstrating great potential for improved IBD therapy (Cristelo et al., 2025). Irritable bowel syndrome with diarrhea (IBS-D) manifests as a chronic bowel dysfunction syndrome in adults, defined by pathognomonic features including visceral hypersensitivity, rectal urgency, postprandial bloating, and altered bowel habit. Eluxadoline (ELUX), a first-in-class enteroactive agent, exerts its therapeutic effects *via* multimodal receptor pharmacology—combining μ/κ-opioid receptor agonism with δ-opioid receptor antagonism—to locally modulate enteric neural signaling and visceral nociception, thereby addressing IBS-D symptomatology through dual mechanisms of bowel motility regulation and pain pathway modulation. Because ELUX has poor water solubility, low dissolution rate, and low oral bioavailability, Md. K. Anwer et al. prepared Eudragit S100-coated PLGA nanoparticles loaded with ELUX to significantly improve the dissolution rate and bioavailability of ELUX (Anwer et al., 2017). Enterotoxigenic *Escherichia coli* (ETEC) is the most common cause of childhood diarrhea, and the colonization factor (CF) and toxin are the main ETEC candidate vaccines. S Nazarian et al. studied the oral immunogenicity of PLGA-encapsulated chimeric proteins containing different CFs and non-toxic toxin antigens, and the results showed that PLGA-encapsulated chimeric proteins can prevent ETEC from adhering and causing toxicity (Nazarian et al., 2014).

## 6 Application of PLGA in the treatment of infection

**FIGURE 4:**
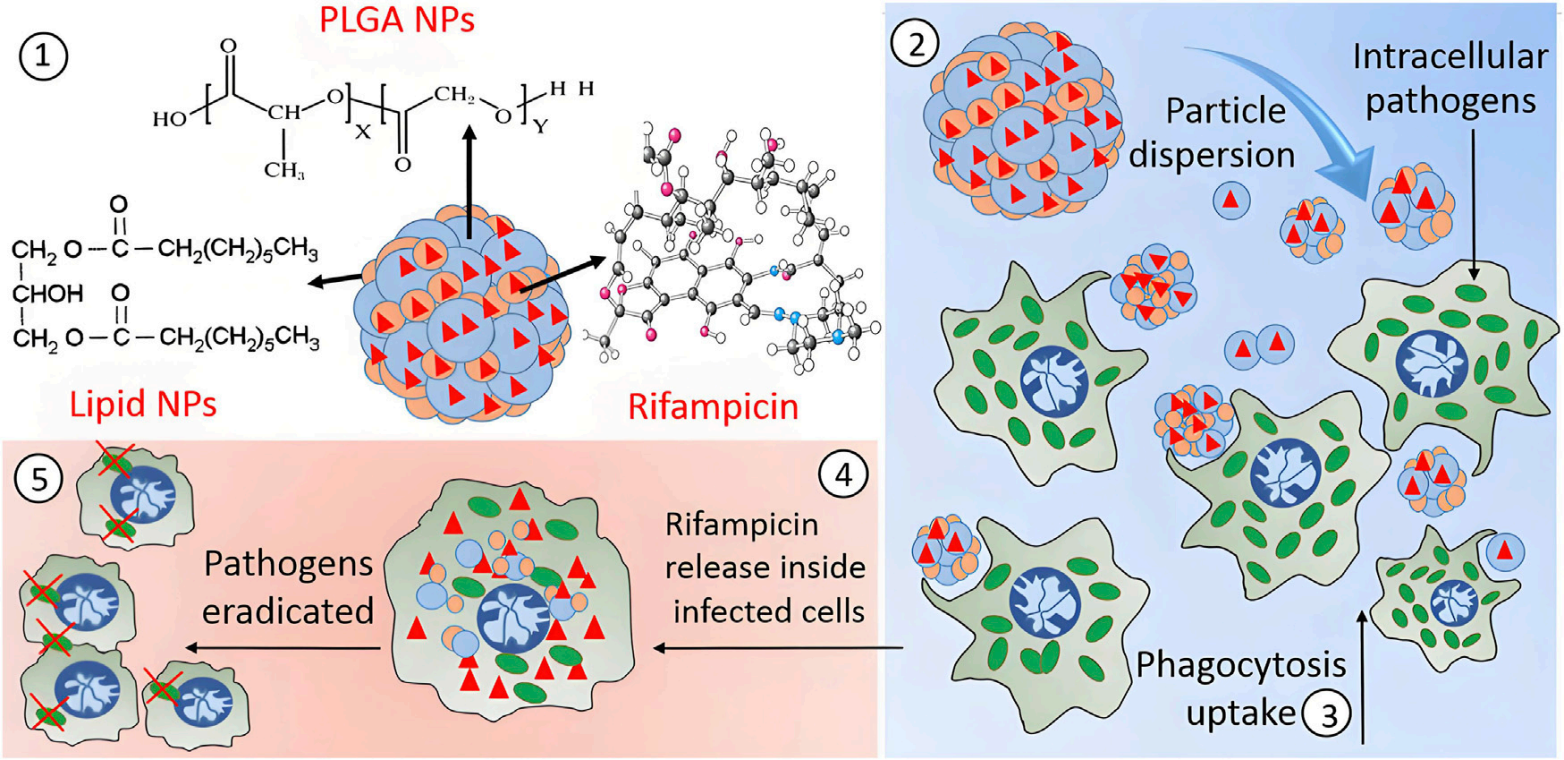
This study encapsulated rifampicin into hybrid microparticles (PLH) composed of PLGA-coated lipid nanoparticles to enhance its inhibitory efficacy against SCV. The spray-dried PLH microparticles loaded the drug within both polymer and lipid phases. Upon decomposition into nano-aggregates, PLH promoted uptake by RAW264.7 macrophages, significantly reduced intracellular bacterial survival, and exhibited no significant toxicity (Maghrebi et al., 2020).

The rise of antibiotic resistance is due to the abuse of antibiotics, which also promotes the development of multi drug resistant microorganisms. If there is no suitable new drug for treatment, the diseases involving drug-resistant bacteria infection will be difficult to control. The escalating global challenge of antimicrobial resistance necessitates the strategic development of precision-targeted anti-infective therapeutics with pathogen-specific activity profiles. Nanotechnology may be one of the best choices, because it can develop intelligent nanoparticles with required properties by using physical and chemical properties, enable site-selective transport of therapeutic payloads to pathologically defined loci with spatiotemporal precision, and reduce the incidence of drug resistance. Because of its good biocompatibility and biodegradability, PLGA has been used to load or package antibiotics, natural products or metal ions for antibacterial treatment (Singh et al., 2018; Cresti et al., 2022; Costabile et al., 2024; Liu et al., 2024). Rifampicin is an antibiotic, which is used to prevent various infections caused by mycobacteria. It has bactericidal effect on both Gram-positive and Gram-negative bacteria. Maghrebi et al. engineered rifampicin-encapsulated PLGA-lipid hybrid nanocomposites to combat intracellular persister phenotypes of *Staphylococcus aureus*, specifically targeting biofilm-associated small colony variants through enhanced intracellular penetration and sustained antibiotic retention, which was conducive to the release of drugs into the cells (Figure 4) (Maghrebi et al., 2020). N-Acetylcysteine (NAC) holds potential for tuberculosis treatment. Kabi Raj Chaudhary et al. developed inhalable lactose-coated PLGA nanoparticles loaded with NAC. They achieved enhanced lung deposition and antibacterial activity against *M. tuberculosis* H37Rv, showing a 4-fold improvement in MIC compared to free NAC(Chaudhary et al., 2025). Antimicrobial peptides (AMPS) have broad-spectrum antibacterial properties. Their antibacterial mechanism is the interaction with the cell membrane of pathogenic bacteria. Because they are easy to inactivate and disintegrate in the acidic gastric environment, their therapeutic application is limited. These limitations can be improved by loading peptides and proteins into nanoparticles. Cruz et al. engineered PLGA nanoparticles encapsulating the novel antimicrobial peptide gibim-p5s9k, conducting comparative analysis of microbiocidal efficacy between the unconjugated peptide formulation and peptide-encapsulated polymeric vectors. They concluded that PLGA nanoparticles loaded with peptide showed higher antibacterial activity against *Escherichia coli*, MRSA and *Pseudomonas aeruginosa* than free peptide, which was suitable for oral administration and could be released to the target area to improve the bioavailability (Cruz et al., 2017). Azithromycin (AZI), a 15-membered azalide-class antibacterial compound, demonstrates reduced efficacy against multidrug-resistant clinical isolates of *Enterococcus faecalis* and methicillin-resistant *S. aureus* (MRSA) due to overexpression of ABC transporter-mediated efflux mechanisms. Abo Zeid’s team developed a nanoencapsulation strategy for AZI using PLGA nanovectors, conducting comparative antimicrobial efficacy assessments between the free drug and nanoformulated counterparts toward these pathogens under standardized CLSI guidelines. The surface-functionalized nanocomposites exhibited a 4–8 fold enhancement in growth inhibition rates compared to non-encapsulated AZI, with MIC values against both strains reduced by 64–128-fold (p < 0.01), effectively overcoming intrinsic resistance phenotypes through bypass of efflux-mediated drug exclusion (Abo-zeid et al., 2022). Platensimycin (PTM), a novel antimicrobial agent targeting the type II fatty acid biosynthesis (FAS-II) pathway, exhibits selective suppression of multidrug-resistant Gram-positive pathogens through competitive inhibition of β-ketoacyl synthase activity (Dong et al., 2017). Liu et al. engineered PTM-encapsulated PLGA nanocomposites to address the suboptimal pharmacokinetic profiles of platensimycin, conducting comprehensive assessments of antimicrobial efficacy across both cellular and mammalian infection models. The nanoformulation demonstrated targeted biofilm disruption efficacy against MRSA, with enhanced bacterial clearance in phagocytic cells and improved survival outcomes in murine systemic infection studies. This delivery system concurrently optimized drug biodistribution parameters while validating FAS-II-targeted therapeutic strategies for combating multidrug-resistant pathogens (Liu et al., 2020). Lactoferrin, a multifunctional siderophilic glycoprotein with intrinsic antimicrobial properties, demonstrates therapeutic potential in managing ocular surface disorders characterized by tear film instability and inflammatory cascades. Amphotericin B remains a gold-standard therapeutic agent for mycotic keratitis, though its clinical translation is constrained by formulation challenges including aqueous instability and premature precorneal clearance *via* nasolacrimal drainage pathways. To solve the above problems, Sammar et al. Loaded lactoferrin and amphotericin B into PLGA-PEG-PEI nanoparticles to improve their pharmacokinetic properties and corneal penetration. Confocal laser scanning showed that the fluorine labeled nanoparticles had good penetrability. Irritation test, atomic force microscope, cell culture, animal test and histopathological analysis showed that nanoparticles had advantages in reducing inflammatory signs and eradicating fungal infection in rabbit eyes, and would not cause any damage to rabbit eyes (Elhabal et al., 2023). The polymeric extracellular matrix formed by microbial consortia paradoxically functions as a protective barrier against exogenous threats, encompassing both immunological defenses and antimicrobial agents. This adaptive survival mechanism significantly contributes to the emergence of multidrug-resistant phenotypes. Traditional antimicrobial formulations are constrained by intrinsic limitations such as suboptimal membrane permeability, hydrolytic instability, and hydrophobic characteristics. This therapeutic impasse necessitates urgent pharmacotherapeutic innovation through either novel antimicrobial entities or engineered delivery platforms. PLGA-based polymeric architectures are positioned as versatile therapeutic vectors due to their rational design capabilities—exhibiting precise drug payload modulation through controlled synthesis methodologies and programmable surface functionalization with molecular recognition motifs. Therefore, PLGA based nanoparticles can be considered as potential candidates to inhibit and destroy microbial growth by improving physical and chemical properties (Stewart and William Costerton, 2001; Cai et al., 2012; Arafa et al., 2019; Shariati et al., 2022; Landa et al., 2024; Takahashi and Moriguchi, 2024). In the future, researchers will focus on the mechanism of action of PLGA nanoparticles loaded with antibacterial drugs *in vivo* to ensure that the design of these polymer nanocarriers is more reasonable. In addition to being used as a carrier of antibacterial drugs, PLGA nanoparticles are also used to prepare vaccine preparations. Some kinds of Leishmania can not stimulate macrophages and cause chronic infection. Therefore, the activation of macrophages is very important for the elimination of Leishmania parasites in the cells. In order to overcome this inhibition and induce innate immune response, Asal Katebi et al. studied the effects of PLGA nanoparticles encapsulated Leishmania soluble antigen (SLA) and TLR1/2 (Pam3CSK4) and TLR7/8 (R848) agonists on the activation of macrophages infected with Leishmania. The results showed that the above PLGA nanoparticles overcame the inhibition of Leishmania infected macrophages. In addition, they increased the intensity and duration of cytokines and iNOS expression, indicating that PLGA nanoparticles had longer availability or delivery time in macrophages. These findings reflected the advantages of PLGA nanoparticles as a therapeutic vaccine preparation (Katebi et al., 2021).

## 7 Application of PLGA in tumor therapy

**FIGURE 5:**
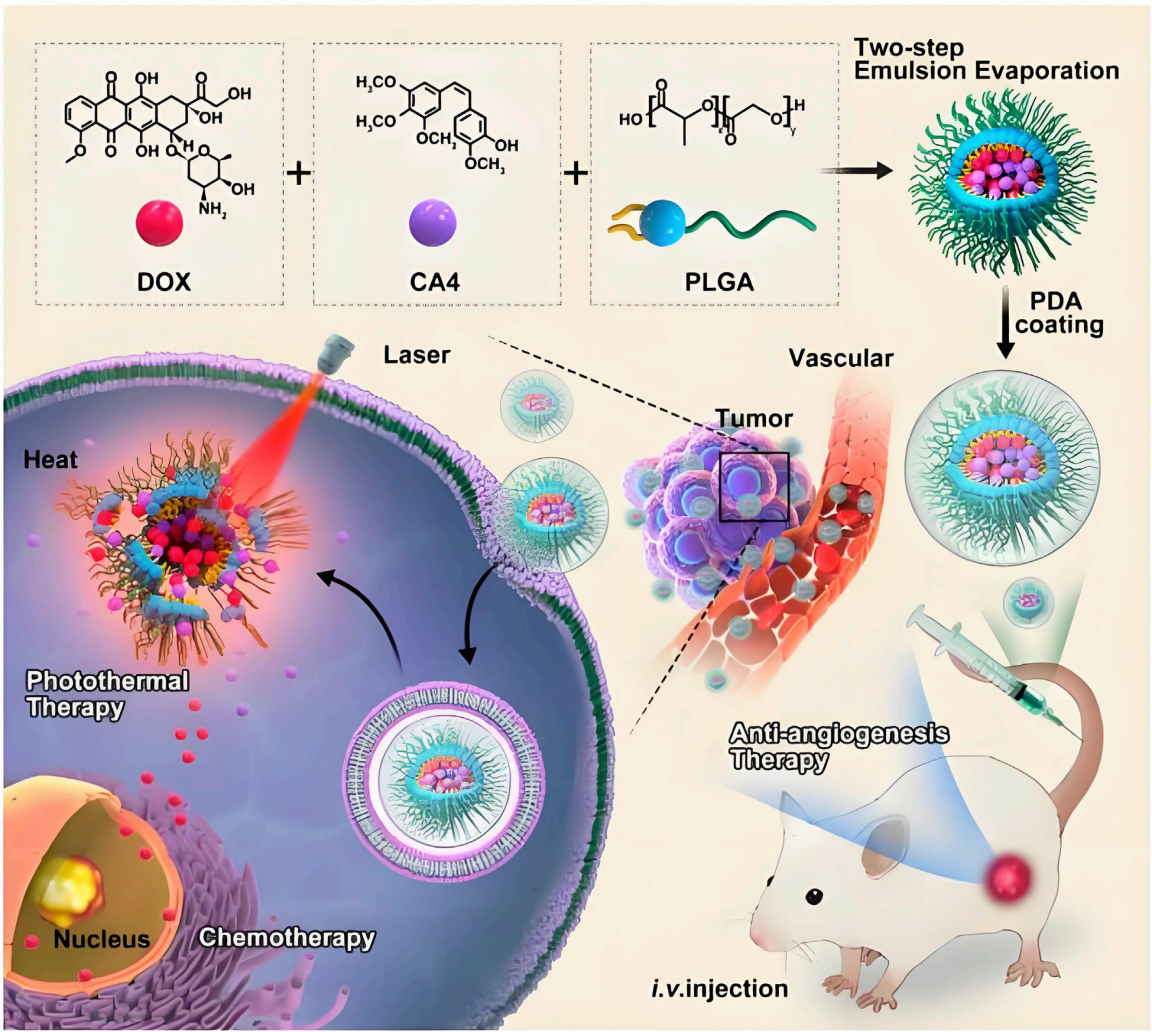
In this study, it combined doxorubicin (DOX) and anti angiogenic drug combrestatin A4 (CA4) into PLGA based co delivery nano hybrid (PLGA/DC NPs) through an improved double emulsification technology, and then modified polydopamine (PDA) on the surface of PLGA/DC NPs by self-assembly method for photothermal therapy (Li et al., 2023).

Malignant neoplasms persist as a predominant contributor to global morbidity and mortality, representing an enduring challenge to population health management (Boire et al., 2024; Holder et al., 2024; Santucci et al., 2024). PLGA based nanostructures have emerged as frontrunner candidates in pharmacological delivery systems, attributed to their programmable physicochemical attributes encompassing modifiable size parameters, enhanced colloidal stability, superior biocompatibility profiles, and controlled metabolic clearance pathways (Dong et al., 2024; Iureva et al., 2024; Sonam Dongsar et al., 2024). PLGA nanocarriers accumulate passively into tumor tissues through the vascular leakage characteristics of solid tumors. The compromised lymphatic clearance mechanisms in neoplastic microenvironments facilitate prolonged intratumoral retention of macromolecular agents, a pathophysiological phenomenon arising from tumor-associated neovascularization anomalies. This tumor-selective biodistribution phenomenon is clinically denoted as the Enhanced Permeability and Retention (EPR) effect, fundamentally driven by angiogenic hypervasculature with defective endothelial fenestrations coupled with lymphatic drainage deficiency in malignant neoplasms (Maeda, 2012; Maeda et al., 2013; Golombek et al., 2018). Strategic surface engineering of PLGA nanoparticles through hydrophilic polymer conjugation (e.g., PEG derivatization) mitigates immune-mediated sequestration by the reticuloendothelial system (RES), thereby extending systemic circulation half-life to potentiate extravasation-mediated tumor accumulation *via* the EPR effect. Building upon this principle, our prior research developed designed PLGA based nanobubbles modified with lipid encapsulating anthracycline chemotherapeutics for EPR-driven tumor-selective delivery. Lipid modification can be used not only as a surface stabilizer, but also as a biocompatible shell of PLGA nanoparticles to avoid non-specific adsorption of plasma proteins and recognition of macrophages. Half an hour after intravenous injection, PLGA nanoparticles were still detected in the tumor area by ultrasound, indicating that lipid modified PLGA nanobubbles have the ability to passively accumulate to the tumor site (Chen Y. et al., 2019). To address *Fusobacterium* nucleatum (Fn)-associated colorectal cancer liver metastasis, neutrophil membrane-coated nanoparticles (NM@PLGA-MTI-OXA) were developed for co-delivery of metronidazole and oxaliplatin. These biomimetic nanoparticles enhance targeted drug delivery, eliminate intracellular Fn, reverse EMT, and remodel the immunosuppressive microenvironment, effectively suppressing tumor metastasis while preserving commensal microbiota (Niu et al., 2025). To augment tumor-specific delivery precision of PLGA nanovectors, surface functionalization with biomolecular recognition motifs (e.g., immunoglobulins, oligopeptides, or low-molecular-weight targeting agents) enables precision recognition of neoplastic biomarkers. This strategic conjugation facilitates receptor-mediated endocytosis through high-affinity molecular recognition events, thereby establishing a dual-targeting paradigm combining active cellular internalization with EPR-driven accumulation (Danhier et al., 2010). Folic acid (FA), a vitamin B9 derivative essential for nucleotide biosynthesis, serves as a low-immunogenicity targeting moiety due to its selective molecular recognition of pathologically upregulated folate-binding proteins. These glycosylphosphatidylinositol-anchored membrane proteins, particularly the FR-α isotype, exhibit dysregulated overexpression on epithelial-derived malignancies. This biochemical specificity has been harnessed in precision tumor-targeting strategies, where FA-conjugated nanovector systems exploit clathrin-dependent cellular internalization pathways to achieve FR-mediated therapeutic payload accumulation in neoplastic tissues (Shen et al., 2012). Ma et al. investigated the biodistribution profile of FA/PEG dual-functionalized PLGA nanocomposites encapsulating indocyanine green (ICG) in FR-α-overexpressing human mammary adenocarcinoma xenografts. Pharmacokinetic analysis revealed enhanced plasma exposure parameters and reduced hepatic sequestration in functionalized vectors compared to unmodified counterparts, demonstrating PEGylation-mediated stealth properties that prolong circulatory persistence while attenuating RES clearance. Tumor-specific biodistribution analysis revealed 3-fold greater accumulation of dual-engineered vectors compared to their unmodified counterparts, demonstrating enhanced tumor-targeting precision. Sustained plasma residence constitutes a critical determinant for passive tumor accumulation *via* the EPR phenomenon. Concurrently, FA-mediated molecular recognition of FR-α overexpressed neoplastic cells facilitated receptor-driven cellular internalization, synergizing passive EPR-mediated deposition with active targeting mechanisms (Ma et al., 2012). Li et al. combined doxorubicin (DOX) and anti angiogenic drug combrestatin A4 (CA4) into PLGA based co delivery nano hybrid (PLGA/DC NPs), and then modified polydopamine (PDA) on the surface of PLGA/DC NPs for photothermal therapy (Figure 5) (Li et al., 2023). The cytotoxic efficacy of polyethylene glycol-functionalized PLGA nanoconstructs encapsulating docetaxel (DOC) conjugated with HER2-specific single-chain variable fragment (scFv) was evaluated in both HER2-overexpressing and HER2-low neoplastic models. Ligand-directed nanoconstructs exhibited preferential necrotic tumor regression in HER2-hyperactive malignancies, thereby validating the therapeutic utility of scFv–DOC–PLGA–PEG as a precision delivery system for receptor-overexpressing carcinomas (Le et al., 2016). Mannosylated PLGA nanoarchitectures were engineered for co-encapsulation of tumor-associated antigenic epitopes and a Th1-polarizing adjuvant (TLR agonist), while simultaneously engaging C-type lectin receptors on dendritic cell populations. This multimodal nanoformulation demonstrated potent antitumor immunomodulation in syngeneic murine B16F10 melanoma models, with significant attenuation of tumor progression kinetics (Silva et al., 2015). It is necessary to inhibit the expression of suppressor of cytokine signaling 1(SOCS1) gene, because it has a negative regulatory effect on the immune response based on antigen presenting cells (APC). M. B. HEO et al. developed PLGA nanoparticles that can simultaneously deliver siRNA and tumor antigen to inhibit SOCS1 gene. PLGA (OVA/SOCS1 siRNA) nanoparticles showed effective uptake by bone marrow-derived dendritic cells (BMDCs), and these nanoparticles knocked out SOCS1 in BMDCs, resulting in the release of pro-inflammatory cytokines including TNF and interleukin (IL-2), which indicated that the system might be an effective method for cancer immunotherapy based on BMDCs (Heo et al., 2014). To circumvent chemoresistance mechanisms, contemporary therapeutic strategies employ RNAi-based gene silencing to abrogate multidrug resistance (MDR) phenotypes through suppression of ABC transporter-mediated drug efflux. Surface-engineered PLGA nanovectors co-functionalized with biotin and MDR1-targeting siRNA were developed for synergistic delivery of paclitaxel payloads, demonstrating enhanced antineoplastic efficacy in both cellular and xenotransplant models compared to conventional chemotherapeutic nanoformulations. The combinatorial therapeutic platform achieved superior tumor growth suppression through concurrent P-glycoprotein downregulation and microtubule stabilization, validating this dual-targeting approach as a paradigm-shifting solution for overcoming transporter-mediated therapeutic resistance (Patil et al., 2010). Preclinical investigations validate miR-145s dual oncoregulatory functions, exerting proapoptotic and antiproliferative activity through cell cycle arrest induction and mitochondrial apoptotic pathway activation across multiple malignancies. Localized delivery of miR-145 *via* PLGA/PEI/hyaluronic acid (HA) ternary nanocomplexes demonstrated enhanced oncosuppressive efficacy in both cellular and orthotopic colorectal carcinoma models, with significant attenuation of neoplastic progression in HCT-116 xenografts. These findings position this nanotherapeutic platform as a breakthrough in RNA-based epigenetic modulation strategies for oncogene silencing therapeutics (Liang et al., 2016).

## 8 Application of PLGA in theranostics

**FIGURE 6:**
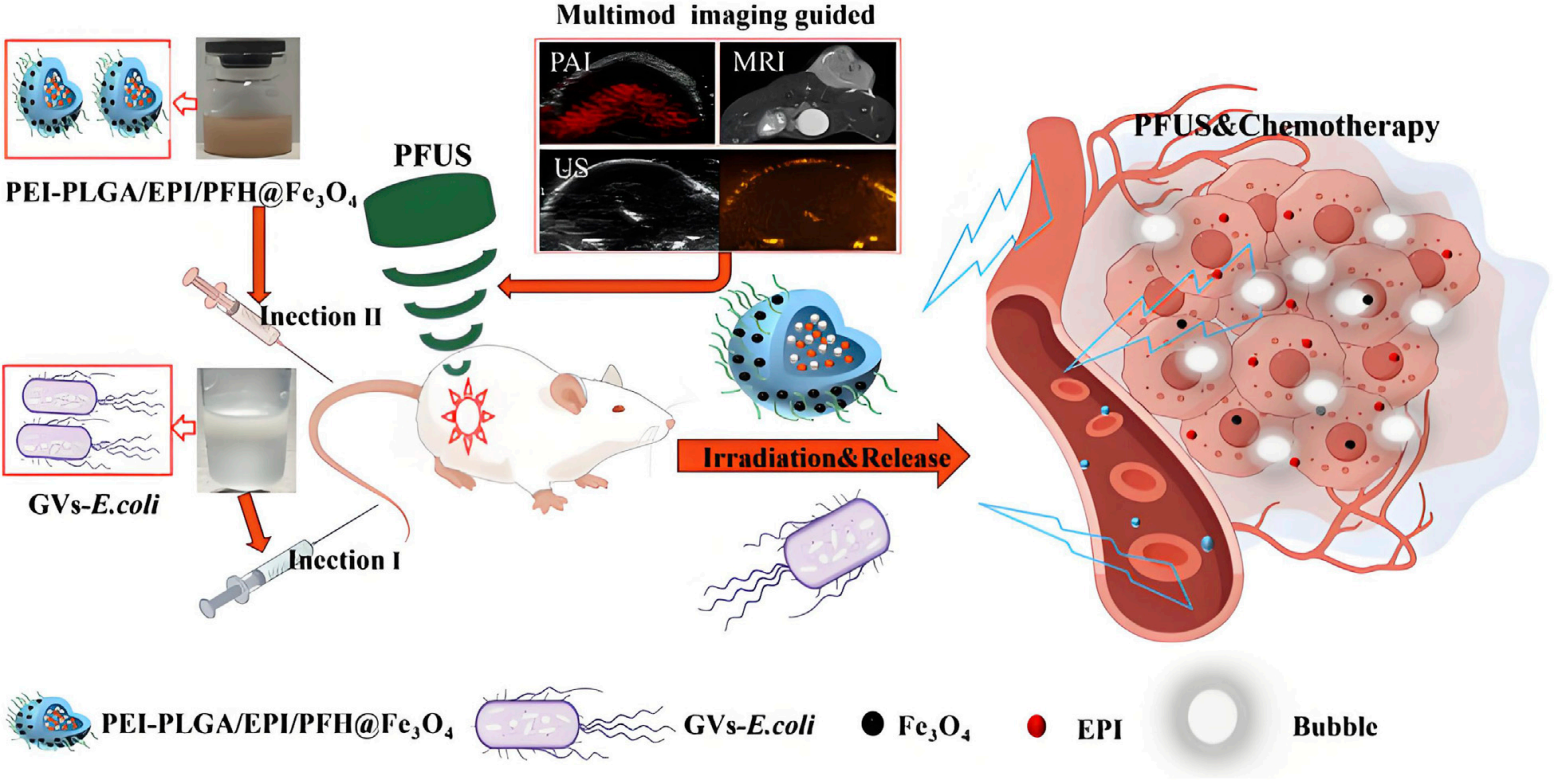
This study developed a gas-producing engineered bacterial synergistic system. The genetically modified *Escherichia coli* (GVs-E.coli) was engineered to carry gas vesicle genes, enabling it to target hypoxic tumor regions while assisting ultrasound imaging and synergizing with pulsed focused ultrasound (PFUS). Concurrently, PEI-PLGA nanoparticles containing Fe3O4, perfluorohexane (PFH), and epirubicin were constructed to integrate chemotherapy and multimodal imaging functionalities. GVs-E. coli mediates the enrichment of nanoparticles in tumor-targeted areas *via* electrostatic adsorption, synergistically achieving ultrasound enhancement, chemotherapy, and tumor eradication (Ren et al., 2024).

Current clinical oncology practice maintains distinct temporal phases for pathological verification and therapeutic implementation. This sequential workflow requiring diagnostic confirmation prior to treatment initiation risks therapeutic window contraction, potentially compromising disease management chronology in neoplastic conditions (Wang et al., 2012). Advancements in molecular visualization technologies have catalyzed the development of integrated theranostic nanoarchitectures, enabling synchronous diagnostic-therapeutic functionality. The strategic convergence of diagnostic probes and antineoplastic agents within tumor-targeted nanovectors permits real-time pharmacokinetic tracking of therapeutic payload delivery to neoplastic sites, while concurrently mapping dynamic tumor progression parameters including volumetric alterations and metastatic dissemination. This closed-loop bioimaging-guided therapeutic paradigm facilitates precision assessment of chemotherapeutic efficacy through continuous spatial-temporal monitoring of tumor response dynamics (Sridharan and Lim, 2023; Wei et al., 2023; Bauer et al., 2024; Speltri et al., 2024). PLGA-based nanostructures have emerged as versatile theranostic platforms in oncological applications, enabling precise tumor visualization through integration with computed tomography, magnetic resonance imaging, and optoacoustic diagnostic modalities. These multifunctional nanoarchitectures demonstrate dual diagnostic-therapeutic capabilities by encapsulating diverse therapeutic payloads including cytotoxic agents, molecular pathway modulators, and photoresponsive compounds, while maintaining compatibility with advanced imaging technologies for real-time treatment monitoring and metastatic surveillance (Figure 6) (Menon et al., 2013; Le Tran et al., 2023; Ren et al., 2024). Yanhua Zhang et al. synthesized multifunctional tumor targeting PLGA-cRGD nanoparticles for dual modality imaging to monitor the therapeutic effect and overcome cisplatin resistance. PLGA-cRGD nanoparticles have the characteristics of clear, targeted, long-term ultrasound imaging and magnetic resonance imaging *in vivo*. PLGA-cRGD nanoparticles under ultrasound can promote the apoptosis of drug-resistant ovarian cancer cells through a variety of mechanisms, including increasing intracellular drug accumulation, reversing the anti apoptotic effect of siBIRC5 and increasing the level of reactive oxygen species (ROS). In tumor bearing nude mice model, PLGA-cRGD nanoparticles have good tumor targeting, high antitumor activity and low systemic toxicity. Therefore, PLGA-cRGD nanoparticles provide a means to monitor the treatment process, and can be combined with ultrasound to overcome the drug resistance of ovarian cancer (Zhang et al., 2021). Perfluorocarbon (PFC) and indocyanine green (ICG) were encapsulated by PLGA for *in vivo* MRI/FLI/PAI multimodal imaging guided photothermal therapy. Then, it was coated with A549 cancer cell membrane (AM) to produce multifunctional therapeutic nanoparticles (AM-PP@ICGNPs). After systemic administration, FLI showed the time-dependent tumor homing of NPs with high sensitivity, 19F MRI provided tumor localization of NPs without background signal interference, and PAI showed the distribution of NPs in tumor. AM-PP@ICGNPs under the irradiation of near infrared (NIR) laser, the tumor cells accumulated in the tumor area showed significant photothermal effect, and enhanced the anti-tumor effect *in vivo* (Li et al., 2020). A pioneering HER2-targeted PLGA-based theranostic platform was engineered to integrate photonic hyperthermia, cytotoxic payload delivery, and diagnostic imaging modalities. The nanovectors incorporated strategic co-encapsulation of zinc phthalocyanine (photosensitizer), Nile blue (optical contrast agent), and irinotecan (topoisomerase inhibitor), with surface functionalization using trastuzumab for precise HER2 epitope recognition. Photothermal characterization revealed intense near-infrared photonic activation capacity, while receptor-specific cellular assays demonstrated 7-fold enhanced molecular recognition specificity for HER2-overexpressing neoplastic populations. Combinatorial photodynamic-chemotherapeutic regimens amplified cytotoxicity 18–20 fold compared to monotherapy approaches. Preclinical validation confirmed dual diagnostic utility in metastatic lesion mapping and therapeutic efficacy, achieving 93% tumor mass regression through spatiotemporally controlled photothermal ablation synchronized with chemotherapy, establishing this modular platform as a transformative precision oncology solution (Komedchikova et al., 2024). Zhiyi Wang et al. developed stimuli-responsive nanoplatforms with tumor microenvironment (TME)-adaptive size modulation and near-infrared (NIR) responsiveness. These hybrid nanostructures utilized a PLGA-based polymeric framework integrated with iron oxide-based core-shell nanocomposites (Fe/FeO@PLGA), encapsulating both chemotherapeutic agents and photoresponsive components. The engineered system exhibited programmable structural reconfiguration—undergoing controlled size reduction and payload release upon NIR activation and acidic TME exposure—while simultaneously inducing oxidative stress amplification through ROS overgeneration. Preclinical evaluations confirmed dual-modal imaging-guided tumor targeting capabilities (fluorescence/MRI) with enhanced therapeutic synergy, validating the platform’s potential for precision oncotherapy through combined chemo-photothermal modalities (Wang Z. et al., 2019). The combination of targeted drug loaded nanoparticles and ultrasound-mediated microbubble destruction (UMMD) can significantly improve cell uptake *in vitro* and drug retention *in vivo*. Lingxi Xing et al. engineered pancreatic adenocarcinoma-targeted PLGA nanovectors functionalized with CA19-9-specific immunotargeting moieties and co-encapsulating the antimitotic agent paclitaxel (PTX). The therapeutic paradigm combines antibody-directed active molecular recognition with UMMD - the former ensuring prolonged systemic retention and tumor-selective PTX release through pH-responsive degradation kinetics, while the latter enhances passive biodistribution *via* EPR effect potentiation and vascular endothelial permeability modulation. *In vitro* mechanistic studies revealed synergistic cytotoxicity characterized by sub-micromolar inhibitory concentrations, G2/M phase blockade, and apoptotic cascade activation, validating this dual-targeting strategy’s capacity to overcome pancreatic cancer’s chemoresistance barriers through spatiotemporal drug delivery optimization. The combination of PTX NPs anti CA19-9 and UMMD has the highest tumor inhibition rate in xenograft tumor of mouse pancreatic tumor. It can promote the pharmacokinetic curve and reduce the clearance rate by increasing AUC, T1/2 and mean residence time (MRT). It can prolong the survival time of tumor bearing nude mice without obvious toxicity (Xing et al., 2016). Shin-Lei Peng et al. designed an active nanoparticles drug delivery system consisting of pegylated epigallocatechin gallate (EGCG), PLGA and iron oxide nanoparticles (IOS). The latter can locate prostate cancer in the molecular image. They proved that nanoparticles can recognize metastasis and enter prostate cancer cells through ligand specificity. The system can enhance the inhibition of cell growth by inducing cell apoptosis. In addition, compared with the systemic combination therapy, the improvement of targeting of nano drugs significantly inhibits the growth of prostate tumor *in situ*. In the presence of iron oxide nanoparticles, low signal intensity of prostate tumor was displayed on T2 weighted magnetic resonance imaging. Therefore, the combination of nano therapeutic particles and molecular imaging system is a promising method for tumor treatment (Peng et al., 2020). To provide a comprehensive analysis of the integration of diagnostic and therapeutic functions in PLGA-based nanosystems, we have compiled a summary of representative theranostic platforms in Table 3, detailing their imaging modalities, therapeutic payloads, targeting strategies, and clinical readiness levels.

**TABLE 3:** Summary of PLGA-based theranostic platforms, their characteristics, and clinical readiness.

Platform name	Imaging modality	Therapeutic payload/mechanism	Targeting strategy	Clinical readiness level	References
PLGA-cRGD NPs	Ultrasound, MRI	Cisplatin, overcoming resistance (increased drug accumulation, siBIRC5 inhibition, ROS elevation)	cRGD peptide (integrin targeting)	Preclinical	Zhang et al. (2021)
AM-PP@ICG NPs	19F MRI, FLI, PAI	Photothermal therapy (ICG)	A549 cancer cell membrane camouflage (homologous targeting)	Preclinical	Li et al. (2020)
HER2-targeted PLGA NPs	Optical imaging (Nile Blue)	Photodynamic therapy (zinc phthalocyanine), chemotherapy (irinotecan)	Trastuzumab (anti-HER2)	Preclinical	Komedchikova et al. (2024)
Fe/FeO@PLGA NPs	MRI, fluorescence imaging	Chemotherapy, photothermal therapy, ROS generation	EPR effect, size-adaptive transformation	Preclinical	Wang et al. (2019b)
Anti-CA19-9 PTX NPs	Ultrasound	Chemotherapy (paclitaxel)	Anti-CA19-9 antibody (active targeting) + UMMD (physical targeting)	Preclinical	Xing et al. (2016)
EGCG-PLGA-IOS NPs	MRI	Apoptosis induction (EGCG)	Ligand-specific targeting (prostate cancer)	Preclinical	Peng et al. (2020)

## 9 Challenges and limitations in multifunctional PLGA nanosystems

The development of multifunctional PLGA nanosystems faces several significant challenges that must be addressed for successful clinical translation. These limitations span manufacturing complexities, regulatory hurdles, and physicochemical stability concerns, each requiring dedicated attention and innovative solutions. (1) Manufacturing challenges and scalability issues: The transition from laboratory-scale production to industrial manufacturing of PLGA-based nanomedicines presents substantial challenges in maintaining product consistency and quality. Batch-to-batch variations and lack of product consistency during scale-up manufacturing remain major hurdles for market launch of polymeric nanocarrier products. Traditional manufacturing methods struggle to maintain the precise control over critical quality attributes such as particle size, polydispersity index, and drug encapsulation efficiency when moving from benchtop to industrial production. To address these challenges, innovative approaches such as inline sonication processes have been developed for industrial-scale production (Operti et al., 2022). (2) Regulatory considerations and standardization hurdles: The regulatory landscape for nanomedicines, particularly multifunctional PLGA systems combining diagnostic and therapeutic components, remains underdeveloped and presents significant challenges. Current regulatory frameworks lack specific guidelines for evaluating the unique properties of nanomedicines, creating uncertainty in the approval pathway. The absence of standardized evaluation systems for nanomaterials and nanomedicines has led to trust crises among manufacturers, healthcare practitioners, and the public regarding product safety and effectiveness. The unique properties of nanoparticles—including surface charge, size effects, and enhanced drug targeting capabilities—create significant characterization challenges for regulatory agencies. These complexities are particularly pronounced for multifunctional PLGA systems that incorporate targeting ligands, imaging agents, and therapeutic compounds in a single platform (Foulkes et al., 2020). (3) Physicochemical stability and biological safety considerations: The stability of PLGA nanosystems presents considerable challenges for both storage and *in vivo* application. Polymer degradation kinetics and drug release profiles can be affected by subtle changes in environmental conditions, manufacturing parameters, and formulation components. Storage stability remains a critical concern, particularly for multifunctional systems incorporating diagnostic agents. However, long-term stability data beyond 1 week remain limited for many multifunctional formulations, especially those integrating imaging agents and targeting moieties. These physicochemical instability issues further exacerbate biological safety concerns. Variations in particle size, surface properties, or premature drug release during storage or after administration may significantly impact immunogenicity, as unstable particles can trigger unintended immune responses. Moreover, aggregation or decomposition of nanosystems could lead to altered biodistribution patterns, increasing the risk of accumulation in non-target tissues and potential systemic toxicity. Uncontrolled release profiles also complicate pharmacokinetic behavior, making it difficult to achieve therapeutic efficacy while minimizing adverse effects. Therefore, comprehensive studies on both physicochemical stability and biological safety, including immunogenicity, systemic toxicity, pharmacokinetics, and biodistribution, are essential to ensure the clinical translatability of PLGA-based nanosystems (Kwon et al., 2024; Liao et al., 2024; Barbery et al., 2025).

## 10 Conclusions and future prospects

PLGA and its copolymer derivatives have been extensively engineered as versatile nanotherapeutic platforms, leveraging their intrinsic biocompatibility to achieve superior clinical translation potential. Preclinical studies across cellular and organismal models consistently demonstrate enhanced therapeutic indices of PLGA-based nanoarchitectures in diverse pathologies. These systems allow precision molecular engineering *via* surface functionalization, such as polyethylene glycol conjugation, albumin corona formation, and molecular recognition motifs, to prolong systemic circulation and enhance site-specific accumulation. Applications extend beyond oncology and cardiovascular disease to include immunomodulatory vaccine delivery and regenerative tissue scaffolding. However, the advancement of multifunctional PLGA nanosystems faces interconnected challenges, including manufacturing inconsistencies that complicate regulatory evaluation and physicochemical instability affecting both production and compliance. To address these issues, the integration of artificial intelligence and advanced imaging techniques offers promising avenues for improved characterization, for example, AI-based image processing and segmentation, already applied to collagen implants by FDA researchers for quantifying porosity, drug location, and release kinetics, could be adapted to PLGA systems for better performance control. Furthermore, the development of physiologically based pharmacokinetic (PBPK) models provides a mechanistic framework to establish in vitro-in vivo correlations for PLGA implants and long-acting injectables, helping to bridge the gap between experimental characterization and clinical outcomes, thereby facilitating regulatory acceptance. In conclusion, despite the considerable promise of multifunctional PLGA nanosystems for theranostic applications, overcoming challenges related to manufacturing scalability, regulatory uncertainty, and stability remains essential. Emerging technologies, such as continuous manufacturing, machine learning, and advanced characterization methods, are critical to realizing the full potential of these innovative nanoplatforms.
